# Correction: Multiple pH responsive zwitterionic micelles for stealth delivery of anticancer drugs

**DOI:** 10.1039/d4ra90139h

**Published:** 2024-11-28

**Authors:** Jin Ma, Ke Kang, Qiangying Yi, Zhirong Zhang, Zhongwei Gu

**Affiliations:** a National Engineering Research Center for Biomaterials, Sichuan University 29 Wangjiang Road Chengdu 610064 China zwgu@scu.edu qyi@scu.edu.cn +86-028-85410336; b West China School of Pharmacy, Sichuan University China

## Abstract

Correction for ‘Multiple pH responsive zwitterionic micelles for stealth delivery of anticancer drugs’ by Jin Ma *et al.*, *RSC Adv.*, 2016, **6**, 64778–64790, https://doi.org/10.1039/C6RA11645K.

The authors regret that an incorrect version of Fig. 10 and 11 were included in the original article. The correct version of Fig. 10 and 11 are presented below.

**Fig. 10:**
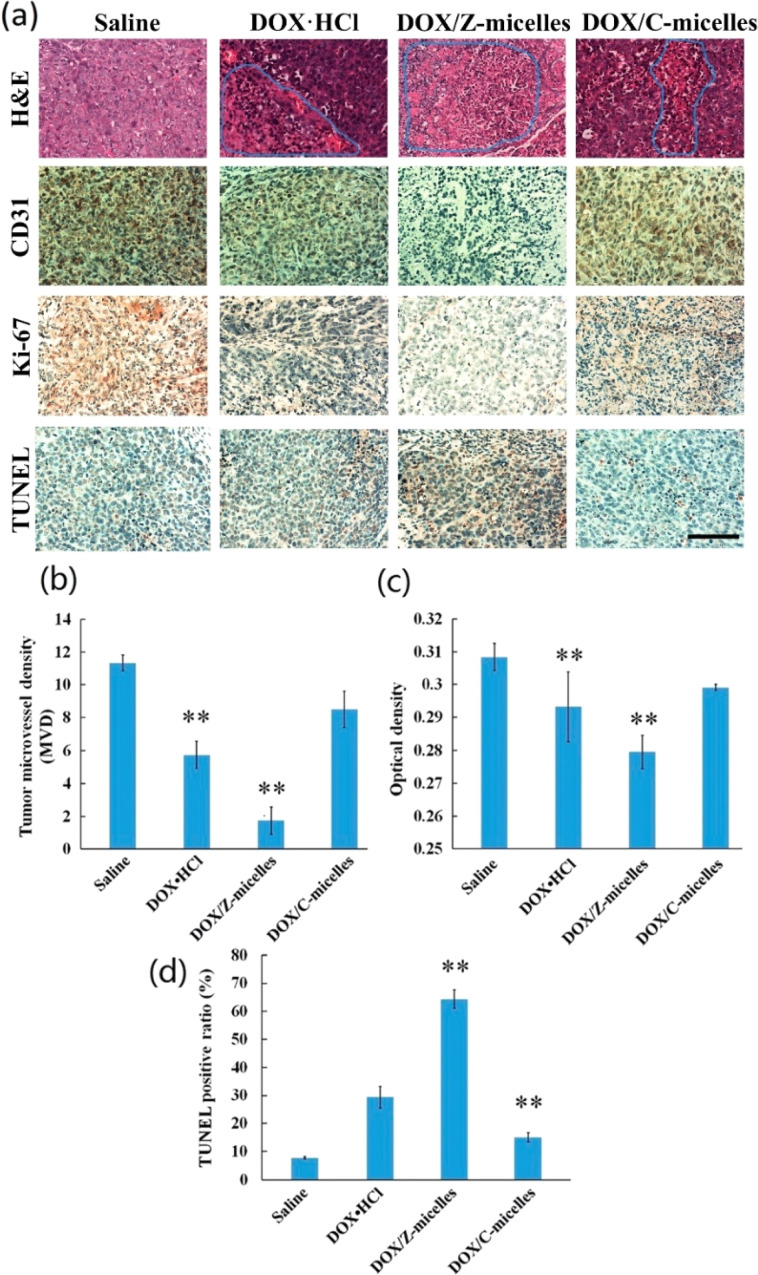
The H&E, CD31, Ki-67, and TUNEL assays for tumor tissues of various treatment groups (tumor: ×400). (a) The apoptosis and necrosis areas labeled with blue in H&E assay. CD31-positive vessels, Ki-67-positive cells and TUNEL-positive were stained brown. Scale bar measures 100 mm. (b) The ratio of CD31-positive to total area of each group was calculated as tumor microvessel density (MVD). (c) Measurement of Ki-67-positive OD (optical density) of each group. (d) The percentage of TUNEL positive cells of tumor tissues in each group (means ± SD, **p < 0.001, compared to saline).

**Fig. 11:**
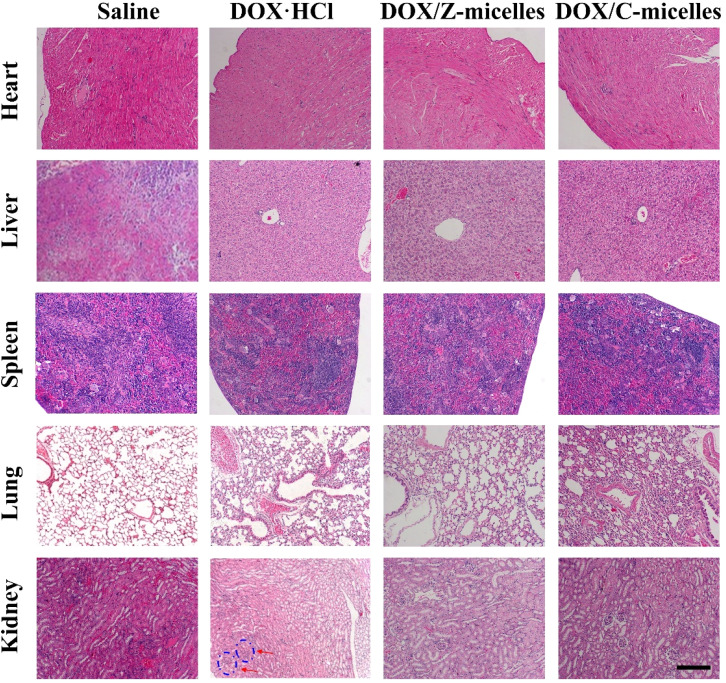
Histological analysis for different organs of tumor bearing mice treated with saline, DOX·HCl, DOX/Z-micelles, DOX/C-micelles. (All tissues: ×100). The mice administrated with free DOX·HCl resulted in kidney glomerular swelling (pointed out by red arrows). Scale bar measures 100 mm.

The authors regret that there was an error in the sentence in lines 23–24 in the right column on page 64787 of the original article. The text originally read “In the H&E assay (Fig. 9a, 1st row), the DOX/Z-micelle group revealed the most apoptosis and…”. This sentence should read “In the H&E assay (Fig. 10a, 1st row), the DOX/Z-micelle group revealed the most apoptosis and…”.

There was also an error in the sentence in lines 29–30 in the right column of page 64788 of the original article. The text originally read “As shown in Fig. 11 (4th row), glomerular swelling was obvious…’. The sentence should read “As shown in Fig. 11 (5th row), glomerular swelling was obvious…”.

The Royal Society of Chemistry apologises for these errors and any consequent inconvenience to authors and readers.

